# Perspectives on DMPA-SC for self-injection among adolescents with unmet need for contraception in Malawi

**DOI:** 10.3389/fgwh.2023.1059408

**Published:** 2023-03-22

**Authors:** Gracious Ali, Chelsey Porter Erlank, Frehiwot Birhanu, Melinda Stanley, Jessie Chirwa, Fannie Kachale, Andrews Gunda

**Affiliations:** ^1^ Sexual, Reproductive, Maternal and Newborn Health program, Clinton Health Access Initiative (CHAI) Inc., Lilongwe, Malawi; ^2^ Analytics and Implementation Research Team, Clinton Health Access Initiative (CHAI) Inc., London, United Kingdom; ^3^ Independent Consultant, Sydney, NSW, Australia; ^4^ Reproductive Health Directorate, Malawi Ministry of Health, Lilongwe, Malawi; ^5^ Clinton Health Access Initiative (CHAI) Inc., Lilongwe, Malawi

**Keywords:** adolescents, self-injection, family planning, contraception, DMPA-SC, unmet need for contraception

## Abstract

**Introduction:**

Malawi has made progress in expanding access to modern contraceptive methods over the last decade, including the introduction of depot-medroxyprogesterone acetate subcutaneous (DMPA-SC) in 2018. DMPA-SC offers women the option to self-inject at home and may benefit adolescents with unmet need for contraception due to its discretion. This qualitative study was conducted to assess perspectives and preferences of adolescents with unmet need for contraception regarding the self-injection option of DMPA-SC in Malawi.

**Methods:**

Six focus group discussions were conducted involving 36 adolescents with unmet need for contraception (aged between 15 and 19 years, married and never-married) in October 2021 in three districts in Malawi. Data were coded inductively and analyzed thematically, using Dedoose software. Two validation workshops were conducted with other adolescents with unmet need in February 2022 to elucidate the preliminary findings.

**Results:**

DMPA-SC attributes such as discretion and reduced facility visits were ranked most appealing by both married and never-married adolescents, particularly for adolescents needing covert contraception use. Concerns about self-injection included fear of pain, injury, and doubt in ability to self-inject. Never-married adolescents had additional concerns around privacy at home if using covertly, and fears of affecting long-term fertility. Overall, health surveillance assistants (community-based healthcare workers) were voted to be the most private, convenient, and affordable sources for potential DMPA-SC self-injection training.

**Conclusion:**

Self-injection of DMPA-SC may offer an appealing option for adolescents in Malawi, aligning most closely to the needs of married adolescents who may wish to delay or space pregnancies conveniently and discreetly, and who also may face fewer access barriers to receiving self-injection training from health care providers. Access barriers including stigma and concerns about privacy at home for adolescents needing to use contraception covertly would need to be adequately addressed if never-married adolescents were to consider taking up this option.

## Introduction

Access to modern contraceptives is an integral part of sexual and reproductive health and rights. Malawi has made great strides in expanding access to family planning (FP) in recent years. The Ministry of Health in Malawi provides free of charge, voluntary FP services and a broad range of contraception methods in all public facilities and at community level through Health Surveillance Assistants (HSAs) and Community-Based Distribution Agents (CBDAs). According to FP2020 tracking reports, the modern contraceptive prevalence rate among all women of reproductive age in Malawi increased from 38.1% in 2012 to 48.9% in 2020, though still falling short of the government's target of achieving 60% by the same year. In addition, the percentage of all married/in-union women with unmet need for modern methods of contraception in the country was also estimated to have decreased from 25.5% to 16.7% during the same period (1). This progress was achieved through expanding access to reliable modern contraceptive methods, including injectable contraceptives, which were estimated to be the most commonly used method in Malawi by 2020, at 49.8% of the contraceptive method mix (2). Injectables are also the most commonly used methods among adolescents aged 15–19 in Malawi (3).

Despite these important gains, adolescents remain marginalized when it comes to full attainment of their reproductive health needs. At a population of 4.7 million (4), which represents about 26% of the total Malawi population of 18 million (5), adolescents aged 10 to 19 years old are an important demographic group. However, child marriage and teenage pregnancy are problems of public concern. A 2021 official joint statement by UNICEF, UNFPA and WHO in Malawi reports that 50% of all Malawian adolescent girls marry by their eighteenth birthday (4). An estimated 29% of 15–19 year old women in Malawi have begun childbearing (6) and Malawi has one of the highest adolescent fertility rates in Eastern and Southern Africa (7, 8). Unmarried adolescents are disproportionately at risk of unintended pregnancy, with low access to reproductive health information and services, when compared to married adolescents. Unmet need for FP among married adolescents aged 15–19 years is at 22% (3) while for never-married adolescents of the same age group this figure rises to 52% (6). This situation greatly contributes to other related health outcomes of national concern such as abortions, which are estimated at 21 abortions per 1,000 female adolescents aged 15 to 19 years (most of which are unsafe), high rates of child marriages and high incidence of HIV infections and sexually transmitted infections (4).

Nevertheless, Malawi's policy framework is generally supportive of adolescent reproductive health. The National Reproductive Health Service Delivery Guidelines, National Youth Friendly Health Services Strategy and National Youth Policy all support a tailored approach to delivering services to adolescents (9–11). In addition, the updated national reference FP manual 2021 states that age is not a medical reason for denying any method to adolescents and young people (1). However, implementation of national policies is often hampered by socio-cultural factors including stigma around pre-marital sex, discrimination from health care providers, negative cultural and gender expectations or norms (12), and negative myths and misconceptions around use of modern contraceptive methods (12, 13).

Depot medroxyprogesterone acetate subcutaneous (DMPA-SC) is a new formulation of the popular intramuscular injectable depot medroxyprogesterone acetate intramuscular (DMPA-IM) but the subcutaneous formulation allows any trained person, including women themselves, to administer it. Thus it provides women the autonomy to self-inject in the comfort of their homes once they have successfully been trained. DMPA-SC, including for both provider-administration and self-injection, was introduced in Malawi in 2018 following a successful randomized controlled trial that was conducted in one district between 2015 and 2017 (14). This study demonstrated a high acceptability rate for use of DMPA-SC “at home” as opposed to in a clinic by a provider (70% preferred this option among injectable users); a high rate of willingness to continue to self-inject (SI) among SI clients (98%); and a high rate of willingness to self-inject in the future among provider-administered (PA) clients (78%). Similarly high acceptability figures have been reported among injectable-experienced populations in other countries, such as Senegal (15) and Ghana (16). Additional studies in Malawi have also provided important insight on clients' and providers' experiences with DMPA-SC, including for SI. Some SI clients described initially feeling apprehensive about SI, mainly because they were nervous or felt doubt about their ability to self-inject; however, all clients interviewed ultimately successfully self-injected (17). The SI option has also been shown to increase continuation rates among injectable clients in Malawi (73% SI clients continuing at 12 months compared to 45% PA clients), including among young women aged between 18 and 26 years (18), with similar results found in studies in other settings (19).

Adolescents are a key focus population for the Ministry of Health's family planning program in Malawi, however to date only a few studies have looked specifically at adolescents' perspectives and experiences with regards to DMPA-SC, including for self-injection. One study in Malawi investigating how young DMPA-SC SI clients managed disposal of their used Unijects, found that 10% of existing adolescent SI clients sampled in Malawi were covert users—something which may affect their ability to dispose of used Unijects at facilities as recommended (20). Another study among DMPA-SC clients in Malawi showed that younger self-injectors (18–24 years old) have similar continuity rates with using DMPA-SC compared to older women (21). It had been hypothesized during the roll-out of DMPA-SC in Malawi that the SI option could be a feasible, discreet and convenient option for adolescents, as indicated by studies from other contexts (22–24), and could even help expand access among adolescents with unmet need for contraceptives. A recent study from Uganda (24) found no significant differences in competence in initial and subsequent SI of DMPA-SC between adolescent self-injectors (aged under 20) and older women, a finding which raises prospects of feasibility of SI among adolescents in similar contexts. However, adolescent self-injectors in the same study were less likely than older women to report convenience and time-saving as reasons to self-inject, were less likely to receive sufficient training to build their confidence to self-inject, and were more likely to be concerned about possible discovery of Unijects at home—and as such, they would likely require additional support from health care providers, family and partners to overcome these challenges.

More evidence was needed on how well DMPA-SC (including the self-injection option) aligned with adolescents' contraceptive needs and preferences in the Malawian context, and in particular among adolescents not already using a modern method. The objective of this study was to explore if and how adolescents who have unmet need for contraception may benefit from DMPA-SC, including the SI option. Specific sub-objectives included:

i. Assessing adolescents' knowledge about the option to self-inject a contraceptive and their perspectives on this option.
ii. Understanding adolescent needs and preferences for contraceptive methods and providers, and how the DMPA-SC SI option might align with those needs
iii. Determining whether expanding DMPA-SC access points to include Youth Centers and CBDAs may influence uptake of DMPA-SC SI among adolescents.
iv. Identifying supply and demand side enablers and barriers that may potentially influence uptake of DMPA-SC SI among adolescents with unmet need for contraception.

## Materials and methods

### Design of study

This study was part of a larger cross-sectional qualitative study that sought to determine the barriers and enablers affecting the uptake and continued use of DMPA-SC for SI in Malawi. The results from the other research questions will be published elsewhere in due course. For the adolescents' component of the study, six focus group discussions (FGDs) were conducted with female adolescents aged 15 to 19 years with unmet need for contraception (i.e., adolescents who are sexually active, currently not pregnant, who wanted to avoid pregnancy in the next 2 years but were not currently using modern contraception). Each FGD included six female adolescents (*N* = 36) and three FGDs were conducted with never-married adolescents while three were conducted with married/in-union adolescents. Data was collected in October 2021.

### Study sites and populations

One district was selected, using simple random sampling, in each of Malawi's three regions: Nkhotakota (Central Region); Zomba (Southern Region); and Mzimba South (Northern Region). Two public facilities with a minimum of ten DMPA-SC clients per month were then randomly sampled per district (six facilities in total). At each of the study sites, adolescents aged 15–19 years with unmet need for contraception (married/in-union or never-married) were purposively sampled from surrounding catchment populations. HSAs working in the communities around the study sites conducted initial recruitment of potentially eligible adolescents before the adolescents were screened against the inclusion criteria by the study team to confirm eligibility. The total number of FGDs was based on theoretical saturation, which refers to the point at which no new concepts emerge from the review of data drawn from a sample that is diverse in pertinent characteristics and experiences (25). In order to assess whether theoretical saturation had been achieved, the study team reviewed initial transcripts from the data collection to assess the variation in themes arising, and made a judgment call based on the information reviewed that the final number of FGDs should be maintained at six as originally planned. Participant characteristics are outlined in the Results section of this article.

### Data collection

**Figure 1:**
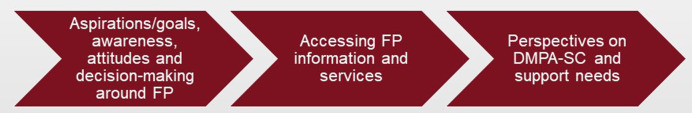
Theoretical behavior change framework.

Recognizing that uptake of any method of contraception requires a behavior change process on the part of the user (26), a theoretical framework (Figure 1) was developed to help better understand where exactly along this behavior change journey towards DMPA-SC SI adolescents with unmet need might face enablers or barriers. This framework was based on findings from the broader literature on FP and DMPA-SC and was used to help develop semi-structured discussion guides with exploratory open-ended questions. As Nkhotakota and Zomba districts are predominantly Chichewa speaking districts, while Mzimba South district is predominantly Tumbuka speaking, study tools were translated into both languages.

The adolescents in the FGDs also engaged in three participatory exercises:

1. **Archetyping**—developing a fictional character “like them” to represent them and their peers, to try and build group trust and encourage open dialogue on the sensitive topic of contraception
  Personas or behavioral archetypes are often used in user-design research (such as human-centered design) to generate a fictional “character” representing one or more different types of users (or potential/target users) of a service or product. Archetypes or personas are typically created by drawing on primary or secondary qualitative and quantitative data about target users to generate the “typical” user profile, their characteristics and expected behaviors (27, 28). However, archetyping can also be used as a participatory qualitative technique within focus groups themselves, leveraging the benefits of a third-person fictional character to whom participants can attribute their own experiences or observations of others' experiences, without leaving themselves vulnerable to judgement from peers within the group (29).
  In this study, participants collaborated to physically draw out an archetype “like them” on paper, guided by prompts from the facilitator. For example, the facilitator asked the groups to name the archetype, share thoughts on her family structure, describe what she does in a typical day, what her dreams are for the future, and who she trusts, etc. Dissent or disagreement about any aspects were noted in analysis, however the groups were encouraged to find consensus on the archetype's identifying features for the purposes of the exercise. The facilitator then referred back to the archetype when posing later questions about contraceptive preferences, experiences and behaviors—for example “How would [archetype's name] feel about this? What would she do?”. As the participants grew more comfortable with the focus group discussions, some participants switched to speaking in first person about their own experiences and thoughts. This explains the switching between third and first person in the subsequent quotes in this article—and when quotes refer to “she” or “her” this indicates the participant is talking about the archetype.
2. **DMPA-SC attributes exercise**—*breaking down the attributes of DMPA-SC and asking about each attribute's suitability for the archetype individually*
  For this exercise, married and never-married adolescents in the focus group discussions were introduced to 12 attributes of DMPA-SC one-by-one and asked if they felt each attribute made the product suitable or unsuitable for their archetype. These attributes were drawn from the broader literature on DMPA-SC features that appealed or did not appeal to women and adolescents in Malawi and other contexts (17, 24, 30) and care was taken to try and use neutral and balanced framing of each attribute. The wording of the 12 attributes of DMPA-SC included in the exercise are listed in Table 1. These include some attributes that would apply to DMPA-SC whether it was self-injected or provider-administered (for example, menstrual changes) and others that were specific to self-injection (for example, use at home). This was a deliberate decision of the study team, recognizing that adolescents would be taking into account both the features associated with self-injection specifically, and DMPA-SC more broadly, when making a decision on whether and how to take up the method.
3. **Service delivery point ranking exercise**—*asking adolescents to rank the various service delivery points their archetype may like to receive SI training from*
  Finally, in the service delivery ranking exercise, adolescents in the focus groups were asked to imagine their archetype wanted to be trained in DMPA-SC for SI, and to imagine that there were four places she could go to be trained: (a). Local hospital/health facility, (b). Community-clinic based Health Surveillance Assistant (HSA) (c). Youth Center and (d) Community-Based Distribution Agents (CBDAs). Note that currently in Malawi, training on DMPA-SC SI is available only from facilities or HSAs, but the Youth Center and CBDA options were added to the exercise to understand these options as comparators.

**Table 1:** Attributes of DMPA-SC introduced to adolescents during focus group discussions.

Attribute	Wording used during focus group
Fewer visits to facility	“Self-injection means a woman only has to visit the facility or HSA once every twelve months, because she can collect multiple doses at once and store them at home”. Does this make it suitable or unsuitable for [archetype's name]?
Use at home	“Self-injection can be used at home, as long as there is a private space.” Does this make it suitable or unsuitable for [archetype's name]?
Discontinue without help	“If a woman decides she wants to stop using self-injection (e.g. if she wants to get pregnant), she can make that decision alone, without asking a provider to help her stop the method” Does this make it suitable or unsuitable for [archetype's name]?
Easy to use	“Self-injection requires one-off training from a provider or HSA. After that, a woman can self-inject on her own easily” Does this make it suitable or unsuitable for [archetype's name]?
Discreet once injected	“Once injected, the hormones stay in the body and cannot be seen or felt by anyone else” Does this make it suitable or unsuitable for [archetype's name]?
Long-lasting	“Once injected, a woman is protected from pregnancy for 3 months, before she needs to inject again” Does this make it suitable or unsuitable for [archetype's name]?
Lighter or no periods	“Most women using DMPA-SC experience lighter or no periods” Does this make it suitable or unsuitable for [archetype's name]?
Effective	“DMPA-SC is highly effective at preventing pregnancy (97%)” Does this make it suitable or unsuitable for [archetype's name]?
Reversible	“If a woman decides she wants to get pregnant and she doesn’t re-inject, she should be able to get pregnant within 12 months” Does this make it suitable or unsuitable for [archetype's name]?
Safe to use while breastfeeding	“DMPA-SC is safe to use while breastfeeding” Does this make it suitable or unsuitable for [archetype's name]?
No need to take action every time you have sex	“Unlike other methods such as condoms, a woman does not have to think about using DMPA-SC every time she has sex” Does this make it suitable or unsuitable for [archetype's name]?
Safe overall	“DMPA-SC may have minor side-effects on a woman's body such as a change in bleeding patterns, but these are not harmful or dangerous” Does this make it suitable or unsuitable for [archetype's name]?

The adolescents were then asked to rank the four options according to four criteria: (a). Privacy, (b). Convenience, (c). Likelihood of judgment and (d). Affordability. These four domains were selected to represent areas of common barriers to contraceptive access (31) that the study team felt adolescents were most likely to be consciously aware of and able to critically appraise for each of the proposed SDP options. The four service delivery options represent a range of different characteristics in these four domains. For example, hospitals/health facilities offer a broad range of contraceptives including DMPA-SC SI to all women, through nurses, midwives, and clinicians. They are generally stand-alone units with almost no ties to the community's individual members and are usually associated with long waiting times and out-of-pocket expenses on transport fares because they are commonly located far from communities.

CBDAs are local volunteers from within the community and responsible for community mobilization for FP and provision of oral contraceptives and condoms through a door-to-door model. They have close ties with each family in the community. HSAs are in the middle between health facilities and CBDAs in terms of level of interaction and proximity with the community. They are a government cadre that provides FP services (including DMPA-SC SI training and resupply) at community level. Both HSAs and CBDAs do not necessarily attract any out-of-pocket expenses because they provide their services right within the community. Finally, Youth Centers are resource centers for youth reproductive health information, and some non-FP services including recreational activities. They are located at a community's central business area and hence may attract some out-of-pocket expenses to access, for example transport fare for adolescents living at a far distance. All staff at these various service delivery points may have received some high-level training on youth-friendly health services integrated into other trainings, but coverage of in-depth youth-friendly trainings tends to be low in practice.

### Analysis

All data was transcribed verbatim from the audio-recorded FGDs and translated into English from the original languages of Chichewa and Tumbuka. Two of the transcripts were then inductively coded by a team of three qualitative researchers separately, in line with the more exploratory nature of the adolescent-specific research question. Alignment and disagreements between codes were discussed by the same team, leading to the development of a final codebook of themes that were then systematically applied across all six transcripts. For some codes, a framework approach was used to facilitate the summary and organization of codes across participants, charting the summarized and coded data into a matrix to allow comparability across FGDs. Data was organized, coded and thematically analyzed using Dedoose software.

Finally, two validation workshops were conducted in Mzimba South in February 2022, where preliminary findings were presented to two different groups of adolescents with unmet need for contraception (married and never-married) to gain their inputs, clarifications, and perspectives on the validity of the results.

### Ethics approval

This study was approved by the National Health Sciences Research Committee (NHSRC) in Malawi, an independent International Institutional Review Board (IRB00003905, FWA00005976), study protocol approval number 21/07/2746.

## Results

### Participant characteristics

The final sample comprised 36 adolescents involved in the FGDs, of whom 18 (50%) were married/in union and 18 (50%) never-married. Their average age was seventeen. Participant characteristics are presented in Table 2.

**Table 2:** Distribution of age and marital status of the study participants.

Adolescents with unmet need for contraception in FGD sample	
**Age**	***N* (%)**
15	5 (14%)
16	6 (17%)
17	19 (53%)
18	4 (11%)
19	0 (0%)
Missing	2 (5%)
**Marital status**
Never married	18 (50%)
Married	18 (50%)

### Adolescents' reproductive aspirations and goals

Adolescents in the focus groups were prompted to describe archetypes “like them” who lived in nearby and had the same marital status as them. Married adolescents were more likely to mention the presence of husbands and close family members (e.g., grandmothers) in their archetypes' lives, while never-married adolescents were more likely to mention the role of their parents and friends in their archetypes' lives. Never-married adolescents mostly felt their archetypes would not yet have children, and may want to delay their first birth, while married adolescents mostly felt their archetype would have at least one child and likely would want another in the future.

The archetypes had dreams and aspirations ranging from investing in their children for a better future, completing school and getting married, or completing school and getting a good job. Married groups tended to mention their archetype having aspirations centered around their children, while never-married adolescents all mentioned their archetypes wanting to finish school before getting married or finding a job:

*“Mercy [archetype] wants to train her children in school so that in the future they should be able to take care of themselves.”—Married adolescents, Mzimba South*

*“Her [archetype’s] dream is to finish school and have a good job.”—Never-married adolescents, Zomba*

Adolescents were very clear about the huge impact of unplanned pregnancy on their lives, particularly if they were never-married. Never-married adolescents spoke of facing a lack of support from parents and stark decisions to make, such as whether to abort the pregnancy, or even commit suicide. Never-married participants felt an unplanned pregnancy may affect their health, cause them to drop out of school, and leave them unable to fulfil their future educational and career plans:

*“If she [the archetype] is in school it means she will have to drop out, because she can’t manage going to school pregnant. It is not possible.”—Never-married adolescents, Zomba*

Married adolescents felt that their archetypes, facing an unplanned pregnancy, would be reassured by support from husbands and from health professionals:

*“If Mercy [archetype] is married and she is pregnant that means there will be no problem because she has a husband who can take care of her”—Married adolescent, Mzimba South*

However, even for married adolescents with social support, it was noted that an unplanned pregnancy could still prevent them from fulfilling future plans.

### Knowledge, attitudes and decision-making around FP

#### Awareness and perceptions of FP

The most frequently mentioned contraceptive method that adolescents were aware of was the injectable, which was mentioned by all groups. The pill, implant and condoms were also mentioned by some groups. When prompted, some participants in both the married and never-married groups revealed they were already aware of a self-injectable contraceptive. However, participants' knowledge was generally high level—that it was possible to inject yourself and that would mean you did not need to go to the facility again for a long time:

*“I heard that with this method doctors/nurses can train a woman to go and do it [self-inject] at home”—Married adolescents, Mzimba South*

Adolescents acknowledged the positives of FP use for birth spacing. However, they were also aware of (and concerned about) the community narratives around side effects of contraceptives, particularly changes in weight and menstrual disruption, as well as the misconceptions about the impact of these side effects on long-term fertility and health. Adolescents with unmet need for contraception typically did not have their own experiences of FP use to compare with the community narratives, and tended to believe the misconceptions they had heard without question:

*“They [contraceptives] might end up destroying their womb and, in the end, not able to have child.”—Never-married adolescents, Zomba*

#### Social support for contraceptive use

Some adolescents indicated that friends could be a supportive influence, while others stated that friends would share stories to discourage them from using FP:

*"In this our community our fellow girls encourage us that we should come and get injected”—Never-married adolescents, Nkhotakota*

*“A lot of girls in this village say they cannot take contraceptive methods, why? When they take contraceptive methods, they say that they are at the same time barring their womb and they will not give birth again.”—Never-married adolescents, Zomba*

All the never-married groups and most of the married groups mentioned not being able to talk about FP with their parents. When asked why, they said parents were hard to approach about such topics, and might shout at them, accuse them of engaging in prostitution, or even potentially throw them out of the house for bringing this up:

*“Other parents can also say, ‘You are asking such so that you can start prostitution?’”—Never-married adolescents, Zomba*

*“They [adolescents] don’t discuss family planning methods with their mothers because they know that their mothers will shout at them… she [archetype] cannot discuss family planning methods with their father fearing that he can shout at her”—Married adolescents, Mzimba South*

There were mixed views from adolescents on what type of support a young woman would get for using contraception from her husband or boyfriend, with some mentioning support, while others said their partners might prohibit them using FP:

*"Some of them encourage their wives that they should use contraceptives, some of them deny their wives to use contraceptives”—Married adolescents, Zomba*

*“…she [archetype] might talk to the boyfriend …[Interviewer: what do they discuss?] like if they have a child, they would want to do spacing so they plan to get family planning method until the child is about 5 years.”—Never-married adolescents, Mzimba South*

Despite acknowledging the influential roles of partners, friends and parents, most adolescents stated that the ultimate decision on the use of FP was theirs:

*"[Interviewer: …who can make the final decision…?] It's Jane [the archetype]. [Interviewer: why do you think it could be Jane? …] Because she is the one who gets the contraceptive and also it is her body that is to get protected.”—Married adolescents, Nkhotakota*

In some cases, where social support could not be relied upon, the participants (particularly the never married participants) acknowledged that this may mean using FP covertly:

*“…as of the woman it is that you have already decided… if the man says no, you are free to go behind his back and do it, that’s all!”—Never-married adolescents, Zomba*

### Access to FP information and services

Adolescents (both married and never-married) stated that the best source of information about contraception is a health facility or hospital. Some married adolescents also mentioned HSAs as a key source. Some (especially those who were never-married) also discussed gaining information about contraception from their peers and others in the community, although views on whether this was a good source of information were mixed.

Most adolescents, both married and never-married, reported that if they wanted to access contraception they would go to a health facility, however some others mentioned HSAs or Social Marketing Organizations as alternate options. When they were asked about places they or their archetypes would avoid getting contraception from, some of the adolescents expressed strong feelings that traditional healers (herbalists) would provide inaccurate advice.

Generally, among the focus groups, ease of access to contraception was thought to be different between never-married and married adolescents, with the former facing low inclination to seek contraception due to fears of being labelled negatively by the society:

*“Participant #3: she [the never-married archetype] might be afraid to come here [the facility]…*

*Participant #1: she might fear being labelled as a prostitute… [Interviewer: if she was married, what would hinder her from accessing family planning methods?] Participant #2: nothing.”—Never-married adolescents, Mzimba South*

### Perspectives on DMPA-SC SI

#### Appeal of DMPA-SC SI among adolescents with with unmet need for contraception

Features from the attributes exercise that all the adolescents felt were suitable for their archetypes included:

• Discreet once injected
• Fewer visits to the facility
• Easy to use, once trained
• Effective at preventing pregnancy
• Long-lasting, 3 months' protection per injection
• Suitable to use while breastfeeding
• No need to take action every time you have sex, like condoms
• Safe overall—usually only minor side effects that are not harmful or dangerous

When asked about which of these features were *most* appealing, many adolescents talked about the reduced visits to the facility and discretion factors, due to the increased privacy and reduced likelihood of being seen by someone they know at a facility.

*“…a lot of girls fail to get contraceptives because they are shy to come to the hospital to get contraceptives as such if they go for this way, they will already have it at home and will be able to inject themselves without being seen.”—Never-married adolescents, Zomba*

Discretion associated with reduced visits to the facility was noted by adolescents to be a particular concern for those who needed to use FP covertly:

*“Yes, they [other adolescents] can be interested with the method because if they use family planning methods secretly that means with this method no one will be able to know that they are using family planning methods”—Married adolescents, Mzimba South*

While there was broad consensus that reduced visits and discretion would be very appealing, some of the attributes of DMPA-SC introduced to adolescents sparked more discussion. For example, the attribute of being able to administer the method at home received mostly positive responses, but some never-married adolescents pointed out that finding a private space at home might not always be easy:

*“…[injecting at home is] not suitable… maybe her [the archetype’s] mother can find her self-injecting in her room, and she might make a mistake (giggles)”—Never-married adolescents, Nkhotakota*

The DMPA-SC attribute of being able to discontinue the method without needing help from a provider also sparked some discussion among the adolescents. Married adolescents felt this was an appealing feature that would help them if they wanted to get pregnant, while some never-married adolescents seemed to feel that it was important to consult a health care provider if they wanted to discontinue FP to get pregnant, seemingly due to a belief that their fertility will have been disrupted or permanently affected by using FP:

*“Considering she [archetype] wants to get pregnant quickly…she is… supposed to go to health counsellor to ask quite well, maybe it might happen that there could also [be] some other complications, they [health counsellor] could help her.”—Never-married adolescents, Zomba*

The DMPA-SC attribute around possible delayed return to fertility also sparked discussion especially among the never married adolescents. During the attribute exercise the adolescents were briefed that it could take up to a year for adolescents discontinuing the method to fall pregnant. Married adolescents felt that a period of up to one year was an acceptable length of time to wait if fertility returned eventually, however never-married adolescents had varied responses. One never-married group felt that a delay in return to fertility of up to one year was positive as their archetype may want to avoid pregnancy as long as possible, while the other two never-married groups felt this might worry their archetypes—because it could indicate an impact on long-term fertility.:

*“…maybe the medicine she was injecting was too strong and she might face difficulties to get pregnant [when she wants to].”—Never-married adolescents, Nkhotakota*

Finally, the attribute that prompted the most discussion and questions was the fact that DMPA-SC use may lead to changes in menstruation, possibly changes in flow, irregular periods or even amenorrhea. Never-married adolescents generally expressed the appeal of lighter, regular menstruation (which they often positively associated with reduced menstrual pain), but mentioned that amenorrhea may make their archetypes worry they were pregnant:

*“Yes [it’s suitable for the archetype], why? Maybe when [she] menstruates she feels stomach aches, so it good that if she gets the injection she won’t be menstruating hence no more stomach pains.”—Never-married adolescents, Zomba*

*“…it [lighter or no periods] cannot be suitable… Because she [archetype] can have thoughts that maybe the medicine didn't work, and she is pregnant.”—Never-married adolescents, Nkhotakota*

When asked if adolescents might have any other concerns about self-injecting, both married and never-married adolescents talked about fear of self-injection going “wrong”, for example the needle not being successfully pulled out after injection and hence ending up getting stuck in the body:

*“Sometimes when administering or injecting the syringe may remain inside the women body and it can cause a lot of problems to her”—Married adolescents, Mzimba South*

One group was aware of the recent stockouts of DMPA-SC at many facilities in Malawi and cited this as a barrier, while another group noted that some adolescents might be shy to expose their thigh to a provider during training. Of all the concerns raised in these discussions, only the concerns about the fear of self-injection going “wrong” (among both married and unmarried adolescents) and the concerns about lack of privacy at home among a minority of never-married adolescents were specific to self-injection.

#### Questions about DMPA-SC SI

When given the chance to ask questions about DMPA-SC SI, all adolescents said their archetypes would want in-depth information on both the way SI works and the pros and cons of the method. Married adolescents tended to ask practical questions, for example whether it was possible to switch methods to SI, how to calculate the date for re-injection, where exactly it could be injected on the stomach, and what the long-term impact of side effects might be. Never-married adolescents' questions tended to focus more on their concerns about side effects and the impact on future health/fertility. One never-married adolescent group asked if it was possible to access DMPA-SC from somewhere outside a facility.

### Preferences for service delivery points for DMPA-SC SI training

Table 3 presents results from the service delivery point (SDP) ranking exercise whereby the adolescents were asked to rank the four potential SDPs for receiving SI training according to privacy, convenience, likelihood of judgement and affordability. Overall, HSAs were voted by the adolescents to be the most private, convenient, and affordable sources of potential SI training. Hospitals/facilities were voted a close second option on privacy and convenience, while both hospitals/facilities and CBDAs were considered roughly equally affordable. However, when asked about the least judgmental option, adolescents felt that hospitals/facilities were the best option, followed by HSAs, while CBDAs and Youth Centers were felt to be the most judgmental.

**Table 3:** Service delivery point (SDP) ranking exercise results.

Attribute				
Rank No.	Privacy	Convenience	Likelihood of judgement	Affordability
1	HSAs	HSAs	Local hospital/health facility	HSAs
2	Local hospital/health facility	Local hospital/health facility	HSAs	CBDAs
3	CBDAs	CBDAs	CBDAs	Local hospital/health facility
4	Youth Centers	Youth Centers	Youth Centers	Youth Centers

In fact, Youth Centers scored poorly on all criteria and across all districts by both married and never-married adolescents, with respondents talking about overcrowding and lack of privacy:

*“(Murmuring) At Youth Centers there are no secrets at all. [Interviewer: There are no secrets in Youth Centers?] Yes (All laugh)”—Never-married adolescents, Zomba*

*“[Interviewer: what’s wrong with the youth center?] it is crowded”—Married adolescents, Zomba*

## Discussion

Adolescents, particularly never-married adolescents, with unmet need for contraception in this study were highly motivated to avoid pregnancy, acknowledging the huge impact an unplanned pregnancy would have on their health, relationships, and future plans. They were relatively knowledgeable about common FP methods and appreciated that FP could help prevent unplanned pregnancy and space births, however there was a lot of concern among adolescents about side effects from hormonal methods and misconceptions about the long-term impact on their fertility (particularly a concern for never-married adolescents) and health (particularly a concern for married adolescents), aligning with findings from other studies in Malawi and similar contexts (13, 32, 33).

Adolescents reported having limited options in terms of people to talk to about contraception, often feeling unable to talk to parents and unsure of whether peers, boyfriends or husbands would provide a supportive environment. All groups clearly expressed that the best and most trusted source of information and services would be a qualified healthcare provider. However, never-married adolescents appeared more likely than their married counterparts to face challenges in accessing that information and care from HSAs/facilities, where fear of being seen by other women at service delivery sites (linked to the stigma around pre-marital sex) was considered a strong barrier in most cases. These challenges echo many of those found to pose barriers to adolescents accessing contraceptive care in other contexts (24, 34) and are not specific to DMPA-SC or self-injection. Indeed, some analyses have found that never-married women are more likely to use easier-to-access methods such as condoms over methods that require interaction with healthcare workers, such as injectables (35), likely due to these sorts of access and stigma barriers. A study with SI users in Uganda also found that adolescents were less likely than adults to first hear about DMPA-SC SI from a community health care worker/Village Health Team member), and more likely to hear about it from friends, suggesting that these access and stigma barriers may also pose a challenge to awareness about the method (24).

Access challenges notwithstanding, adolescents across the focus groups ranked HSAs and then facilities as the most private, convenient, and affordable sources of care, suggesting that any strategy to raise awareness of contraception and DMPA-SC SI among adolescents could focus on these service delivery points in the first instance. This also agrees with other literature which suggests that efforts to increase contraception uptake are likely to be successful when integrated into the routine FP service delivery system (taking a systems approach) as opposed to stand-alone programs (31). However, it was clear in this study that HSAs were not ranked by adolescents as the least judgmental option for care. The reasons for this perspective were not fully explored in this study, but findings from another study with providers in Uganda may provide hints. In that study, health care providers exhibited non-youth friendly attitudes regarding adolescents' use of DMPA-SC SI citing age, parity and competency concerns (30). If similar attitudinal barriers are found to exist among HSAs in Malawi, this may potentially pose a provider-induced barrier to SI uptake among adolescents and therefore suggests that some values clarification and attitude transformation work could be considered to increase youth-friendliness among any HSAs found to be displaying these negative attitudes.

Youth Centers were ranked poorly by the adolescents in this study and were generally felt to be judgmental, crowded and not confidential. This is despite considerable government investment into the Youth Center model and also despite adolescents and parents calling for more separate spaces for youth FP information and services (36). However, this finding aligns with documented global evidence that Youth Centers are not the most effective model for increasing contraceptive use among adolescents (31, 37, 38). These combined findings suggest that expanding access to family planning methods such as DMPA-SC at Youth Centers is unlikely to be effective in encouraging uptake of modern methods among adolescents with unmet need in Malawi.

Some (but not all) of the adolescents in the FGDs felt that DMPA-SC SI could be a suitable method for their peers, citing factors such as discretion and reduced visits to the facility as key features that appealed. However, the strength of the appeal of these factors may depend on contextual factors—a recent study among self-injection users in Uganda found that adolescents were less influenced than adult users by convenience and time-saving when choosing self-injection, and more likely than adults to choose SI because it meant learning a new skill or because their peers recommended it (24).

The strength of the appeal of DMPA-SC and the self-injection option also needs to be weighed against the strength of concerns expressed by adolescents. In this study, married adolescents had fewer concerns about using DMPA-SC SI than never-married adolescents, and these mainly echoed the concerns expressed by older clients in the broader literature (for example, fear of SI, doubt about their own ability to SI, concerns about DMPA-SC side effects). It is important to note that World Health Organization recommends that adolescents aged between menarche and age 18 can generally use DMPA-SC (MEC category 2) (39). Therefore, adolescents' concerns could arguably be addressed through evidence-based quality counselling and good SI training to build client confidence. Never-married adolescents in this study also shared some of the same concerns as their married counterparts, but also had additional worries—in particular whether they would have a private space at home to store and re-inject the method; misconceptions about impact of side effects on long-term fertility; and wanting more reassurance about discontinuation of the method and return to fertility. The strength of these concerns among never-married adolescents appeared to somewhat outweigh the appeal of the discretion and fewer visits to facilities—suggesting that these areas would need to be adequately addressed in any future program aiming to expand access among this group. These findings echo insights from similar studies in Uganda, where fear of lack of privacy at home and misconceptions about the impact of hormones on long-term infertility were cited as key adolescent concerns with the DMPA-SC SI concept (23, 24, 30). The need for a private, safe space at home to take up the SI option may also indicate that for some adolescents, receiving injectables from community-based providers may be the more discreet option.

Overall, the perspectives and preferences of adolescents in Malawi with regards to DMPA-SC are:

*Appeal of DMPA-SC SI* **-** DMPA-SC SI attributes (particularly reduced visits to facilities and discretion) aligned well with the contraceptive needs of married adolescents in Malawi but less well with the needs of never-married adolescents due to concerns about covert use, return to fertility, and misconceptions about impact of hormonal FP use on long-term infertility.

*Concerns about side effects***—**in addition to counselling/training in SI to build confidence, both married and never-married adolescent groups would need good counselling on side effects to address their misconceptions about the long-term impact of hormonal methods on health/fertility.

*Hypothetical preferences for care***—**Adolescents said that HSAs were the most private, convenient and affordable sources of care, while facilities were the least judgmental. Married adolescents appear able to access contraceptive care through the same channels as older women without significant challenges. However, never-married adolescents are still likely to face stigma barriers in access, which would need addressing through youth-friendly training for health care providers and other evidence-based interventions.

### Validation workshops

In February 2022, validation workshops were held with Reproductive Health Directorate colleagues from the Malawi Ministry of Health and two separate groups of adolescents (married and never-married) in Mzimba South. The adolescents were recruited in a similar manner as the ones in the main FGDs and using same inclusion and exclusion criteria. However, the discussions were held in a workshop format—with a team of 4–5 researchers presenting summarized results for input and reactions—and the participants' inputs noted down. In most cases, participants agreed with and validated the preliminary findings from the study. However, a few discussion points arose. Adolescents in the workshops (even never-married adolescents) were generally less concerned about lack of privacy at home than the adolescents in the original sample, feeling they could find secret places to store and use Unijects in their bedrooms. While the limitations of the workshop format mean this new information should arguably not be given the same weight as the more systematically-collected FGD findings, this discussion point could suggest the nature and extent of storage and use of DMPA-SC units at home as a barrier for never-married adolescents could differ depending on individual home circumstances. The adolescents in the validation workshops also felt that facilities were preferable for accessing contraceptives over HSAs in some contexts, depending on how they personally felt about their local HSA. Married adolescents in the validation workshops also felt that their parents would be more supportive of them using FP than married adolescents in the original sample, because their parents had seen the impact of previous unintended pregnancies on their daughters' lives. Given the limitations of the workshop format and lower quality of this evidence, the original study results have not been amended in light of this feedback, but these key discussion points are noted here as a point for reflection that perspectives on DMPA SC, and the SI option, might differ among adolescents depending on individual and contextual factors. The validity, nature and scale of such variation could be explored in future research.

### Study strengths and limitations

The qualitative nature of this study and the inclusion of participatory exercises allowed for a detailed and nuanced understanding of the perspectives of adolescents with unmet need for contraception on DMPA-SC and the self-injection option. The focus group format for adolescents with unmet need for contraception allowed broad perspectives and insights into interactions between adolescent peers. However, there were some drawbacks in that some adolescents reportedly felt uncomfortable to share personal concerns or views in a group. The IRB-mandated requirement for parental consent for adolescents aged between 15 and 17 to participate in the focus groups did not seem to unduly bias the adolescents agreeing to participate (in fact over 80% of the sample were aged under 18), however it may have influenced these adolescents' comfort participating in the discussions on this sensitive topic. Adolescents were reassured of the confidentiality of participation and the study tools were framed to ask about “a typical adolescent” (the archetype) rather than asking personal questions to try to mitigate this risk. The data collectors and supervisors also took steps to make the interviews as accessible as possible by allowing for flexible times and meeting locations, as well as ensuring that the FGDs were held in discreet locations with audio-visual privacy.

Adolescents were screened against the study inclusion criteria of age (15–19 years old), marital status (either never married or married/in-union), sexual activity (had sex at least once), pregnancy status (not currently pregnant), non-use of modern contraceptives (not currently using anything or only using a traditional method to prevent pregnancy), and fertility intentions (desire to delay first/next pregnancy for at least two years). Of this information, only marital status was used for subgroup analysis, and further nuance between other sub-groupings (for example, by parity) was not possible due to the small sample size, but could be addressed in future research.

The use of HSAs for initial recruitment of adolescents may have influenced the profile of the sample in unknown ways. While the vast majority of adolescents screened by the study team were eligible and consented to participate, it is not known how many adolescents declined to participate when approached initially by the HSAs. Finally, the relatively small overall sample (*N* = 36) of adolescents from only three districts means the conclusions from this study may not be generalizable to all adolescents with unmet need for contraception in Malawi.

### Future research

We recommend that further research should be conducted in Malawi and other contexts to explore the experiences of adolescents already self-injecting, especially those who are never-married, who face the highest barriers. Building on the work of Corneliess et al. (24) in Uganda, future studies could focus on understanding adolescents' experiences with the method and level of satisfaction, and how they have managed to overcome challenges around access, storage and use of SI at home (particularly if they use FP covertly) that have been highlighted by their peers with unmet need for contraception in this study. Future research could also monitor and evaluate adolescent and youth uptake of DMPA-SC in the context of a full contraceptive method mix in Malawi and other contexts to determine effective models of care for reaching this population with a comprehensive contraceptive method mix to reduce unmet need for contraception.

## Conclusion

Married adolescents with unmet need found most attributes of DMPA-SC SI appealing and had relatively fewer concerns than their never-married counterparts, suggesting they may consider using SI to meet their contraceptive needs—with comprehensive counselling and training to address their practical concerns and build confidence with the SI option. Married adolescents are likely to be able to access SI training through existing service delivery points (i.e., HSAs and facilities) due to the lower risk of stigma for this group.

While the discretion of reduced facility visits greatly appeals to never-married adolescents, they have significantly more barriers and concerns to overcome before they are likely to take up DMPA-SC SI. They will likely require discreet, convenient sources of SI training, additional counselling time from non-judgmental providers to thoroughly address misconceptions and concerns about fertility, tailored counselling messages, and advice on how to store and re-inject DMPA-SC if they do not have a safe space at home. Where such adolescents are interested in injectables for reasons of discretion from family/partners first and foremost, they should be offered both the self-injected and provider-administered options so they can decide which option is most discreet for their personal circumstances.

For adolescents (particularly married adolescents) considering DMPA-SC SI, HSAs or facilities are likely to be the most private, convenient and affordable source of SI training. However, the fact that adolescents did not rank HSAs as the least judgmental option suggests that some values clarification and attitudes transformation may be needed among HSAs if this cadre is to be considered the vehicle for future efforts to expand access to FP among never-married adolescents with unmet need.

Youth Centers are unlikely to be a preferred service delivery point for contraceptive services by adolescents. Given the relatively low ranking of CBDAs by adolescents in this study, a review of the model may be needed to identify and address existing barriers and bottlenecks hindering youth-friendliness if CBDAs are identified as a potential vehicle for future efforts to expand community access to FP methods such as DMPA-SC among adolescents with unmet need.

## Data Availability

The fully anonymized data supporting the conclusions of this article will be made available by the authors, upon request.
